# Notes of Prof. S. J. Jones’ Clinic at Illinois Charitable Eye and Ear Infirmary

**Published:** 1880-02

**Authors:** F. C. Schaefer

**Affiliations:** Asst. Aural Surgeon


CHICAGO MEDICAL

JOURNAL & EXAMINER.

Vol. XL.—FEBRUARY, 1880.—No. 2.

[In these pages dimension and weight are expressed in terms of the Metric System, and temperature in degrees of the Centigrade Scale.]

### Clinical Lectures.

### Article I.

Notes of Prof. S. J. Jones' Clinic at the Illinois Charitable Eye and Ear Infirmary. Reported by F. C. Schaefer, m.d., Asst. Aural Surgeon.

*Eczema.*—This little girl aged seven years, has an unsightly eruption extending from the concha into the external meatus. It forms a scab or crust on the skin from which there escapes a thin yellowish fluid. The integument in the vicinity of the eruption is rendered highly sensitive by reason of the hyperæmia occasioned by this morbid condition, which is accompanied by more or less pruritus. The itching sensation is sometimes so severe as to lead the child to seek relief by rubbing the surface with the finger, and thus produce friction which only intensifies the process of inflammation, and in some instances produces a diffuse inflammation involving the entire meatus. Upon inquiry, the child's mother states that the eruption has existed three months, that a number of little watery "blisters" first appeared, which soon run together and formed this mass of incrustation, which has probably been thickened by the addition of considerable dirt. This is the usual history of eczema of the ear.

Eczema is classed among the vesicular skin diseases, of which there are three grades, the acute, sub-acute and chronic. The acute is the most common form met with in this locality, and occurs usually in children suffering from defective nutrition, in those who are poorly fed, and in the large class who have inherited a strumous diathesis. It is sometimes brought on by uncleanliness or by the pernicious habit of picking the ears. Occasionally it appears in the adult, resulting from the application of irritating substances to the part involved.

The treatment will vary with the grade of the affection. In the acute form local sedatives are indicated. If accompanied by a burning sensation with considerable tendency to inflammation, mild astringents should be applied to the part.

The sub-acute grade will call for about the same treatment, with the addition of moderate stimulation. In the chronic form emollients are useful to soften the scales, and more decided stimulating applications are recommended. Frequent lubricating of the parts with vaseline to prevent drying and hardening of the secretions, and to protect the diseased parts from the irritating effects of the atmosphere, will be found to be servicable. Constitutional treatment is often required in these cases.

*Acute diffuse inflammation of the external meatus.*—This patient states that there has been an intolerable itching sensation in her right ear during the last two weeks, but two days since this gave way to an intense aching pain, and feeling of fullness accompained by noises in the ear, and that her hearing became suddenly impaired. Upon inspection the external auditory canal is found almost closed by swollen integument. The tragus is thickened and the entrance to the meatus is rendered almost impervious by the tumefied condition of the tissues. These parts are very sensitive to the touch. It would be impossible to obtain a view of the membrana tympani without causing a great deal of suffering to the patient as the thickened walls of the meatus encroach upon the caliber of the canal and would have to be violently pressed apart in order to introduce a speculum, a proceeding uncalled for, and that would aggravate the disease.

The entire internal surface of the meatus is very sensitive and reddened by inflammation. Could a view of the membrana tympani be obtained, its outer surface would doubtless present a somewhat similar appearance.

This is a case of acute, diffuse inflammation of the external meatus, which can be promptly relieved by judicious treatment. Should it be neglected or improperly treated, the inflammation might pass into the suppurative stage and involve more or less destruction of tissue, macerating the membrana tympani and sometimes producing perforation of it, or the acridity of the pus by reason of its irritating qualities, might give rise to the formation of furuncles or polypi. Not infrequently, a low, chronic form of suppurative inflammation supervenes, with an exceedingly offensive discharge, bringing the tissues into a low state of vitality favorable for the lodgment, upon their surfaces, of a parasitic fungus, aspergillus, of which three species have been found in the ear of man, viz: aspergillus flavus, a. glaucus, a. nigricans.

In another class of cases the acute symptoms gradually merge into a chronic inflammation, which has for its sequelæ the suppression of the function of the ceruminous glands and the destruction of the vitality of the epidermis owing to the long continued dry condition resulting ultimately in its desquamation. Frequently a cast of the meatus is thrown off and sometimes exostoses develop. The pain accompanying the acute inflammation is very severe. The explanation for this is to be found in the fact that the integument lining the external auditory canal, in its lower part, lies in such intimate contact with the bone as to almost make it a periosteum, and an inflammatory process here must be similar to a periostitis, which is always extremely painful on account of the compactnesss of structure which is surrounded by bony walls that cannot yield to pressure.

The cause of this condition can frequently be traced to a habit of picking or rubbing the ears with hair-pins and ear-scoops that many people resort to in the belief that proper personal care requires that they should do so, or, as is at times done, to relieve itching in the ears. Ear wax is a natural secretion and serves a useful part in the economy, by keeping the parts pliable and preventing the admission of insects. In health, the ears require no special care.

A prolific cause of this inflammation is the instillation of various oils and ear drops of an irritating character for the relief of ear-ache.

Eczema sometimes is an active cause of diffuse inflammation of the meatus.

Violent efforts made to remove impacted cerumen or foreign bodies from the auditory canal will be followed by acute, diffuse inflammation. It may also be occasioned by inflammation extending from the middle ear in case of suppurative inflammation of that part.

Treatment at first should be antiphlogistic. Scarify the inflamed surface, or apply leeches to the tragus, for at this point the vessels which supply the canal and outer layer of the membrana tympani are more surely reached. In addition to this, advise warm pediluvia and cathartics. A continuous stream of warm water is to be directed into the meatus, or in lieu of this, the meatus, if yet pervious, may be filled with warm water containing morphine in solution ; the application to be frequently repeated and retained with a plug of cotton.

In the chronic form of the disease, thorough cleanliness is to be enjoined and the parts lubricated with vaseline. Should there be pain present, vesication over the mastoid process will be advisable. The suppurative cases are to be treated in the same way with the addition of astringent applications frequently applied, after frequent and thorough cleansing of the parts.

The external meatus, as a consequence of disease, is usually quite sensitive and patients frequently ask the question, what shall be done to protect their ears from cold. As a general thing little, if any, precautionary measures need be resorted to. The meatus forms a natural angle that protects its inner portion and the membrana tympani from the more dangerous effects liable to be produced by the vicissitudes of the atmosphere, yet much comfort may be given the patient by lubricating the meatus with vaseline or diluted glycerine to lubricate the parts and to supply the place of ear wax, the secretion of which has been suppressed by the inflammatory process, and to allay accompanying itching of the meatus. Fifteen grains of carbolic acid dissolved in an ounce of rose-water will aid greatly in allaying itching and destroying the growth, if aspergillus be present, and the meatus be filled twice a day with the solution or moistened with it at shorter intervals.

The very common practice of filling the external meatus with cotton wool on all occasions is objectionable.

As the circulation of the blood in many of the diseases of the ear is defective, exposure of the affected part to a low temperature of the atmosphere, or to high winds may produce discomfort or otherwise do harm. Slight protection to the ear while so exposed may be advantageous, but indiscriminate muffling of the external ear is likely to do more harm than good.

For therapeutic use, where cotton is required, this which has been recently added to the armamentarium of the aural surgeon under the names of borated cotton, iodized cotton, and absorbant cotton, will frequently be found to be both convenient and serviceable.

---

THE late *concours* at Rush Medical College, for the purpose of filling the position of Lecturer on Gynecology in the spring faculty, was rewarded with a large success, considering the number and standing of the competitors and the general excellence of their public efforts. The auditorium of the upper college lecture-room was well filled on each evening of the course, by an attentive congregation of medical gentlemen and students. The position was awarded to Dr. R. Stansbury Sutton, of Pittsburgh, who, it is understood, will make his residence hereafter in this city. Among the most effective of the lectures delivered were those of Dr. A. J. Stone, of St. Paul, Minn., and of Dr. D. T. Nelson, of Chicago. Dr. Stone's candidature was practically annulled by his announced inability to remove to Chicago for the purpose of attending to the duties of the position, but his lecture was one which reflected the greatest credit upon himself, and which produced a most gratifying impression upon the professional gentlemen who were so fortunate as to hear it.

